# Estradiol induces HOTAIR levels via GPER-mediated miR-148a inhibition in breast cancer

**DOI:** 10.1186/s12967-015-0489-x

**Published:** 2015-04-25

**Authors:** Sifeng Tao, Haifei He, Qiang Chen

**Affiliations:** Department of Surgical Oncology, Second Affiliated Hospital, School of Medicine, Zhejiang University, 88 Jiefang Road, Hangzhou, 310009 China

**Keywords:** HOTAIR, Estrogen, miR-148a, Breast cancer

## Abstract

HOTAIR plays an important role in the regulation of cancer cell proliferation and cancer invasion in breast cancer. The up-regulation of HOTAIR has been reported in both estrogen receptor (ER) positive and triple-negative (TN) breast cancer. It has been reported that HOTAIR is regulated by estrogen (E2) via ERs in ER-positive breast cancer. However, it is unknown how HOTAIR is regulated in TN breast cancer. In this study, we found that HOTAIR was increased in the peripheral blood mononuclear cells and cancer tissues from breast cancer patients, and was especially higher in patients with metastatic breast cancer. In addition, we found that estrogen promoted HOTAIR through its receptor GPER and estrogen-induced breast cancer cell migration was reversed by deleting HOTAIR in TN breast cancer cells MDA-MB-231and BT549. Furthermore, we identified that E2-GPER induces the level of HOTAIR through the suppression of miR-148a. miR-148a level was negatively correlated with HOTAIR level in breast cancer patients. After the mutation of the predicted miR-148a binding sites in HOTAIR, miR-148a had no effect on HOTAIR. In conclusion, our findings offer important new insights into the ability of estrogenic GPER signaling to increase the HOTAIR level by inhibiting miR-148a in breast cancer.

## Introduction

Breast cancer is one of the most common malignant diseases in women. However, the molecular pathogenesis of breast cancer remains poorly defined due to its heterogeneity [1]. Despite advances in the treatment of breast cancer, the effective control of metastasis remains a complex problem. It has been reported that over 90% of the deaths of cancer patients are caused by metastasis, which is formed by the spread of disseminated primary tumor cells to distant anatomic sites [2]. Finding new modalities to treat patients who do not respond to conventional treatments has become increasingly important.

Non-coding RNA has become the focus of “next generation” biology. Non-coding RNA includes microRNAs (miRNAs) and long non-coding RNAs (lncRNAs). Roles for miRNAs have been demonstrated in the regulation of a broad range of biological activities and diseases [3,4]. More recently, thousands of lncRNAs, which are transcribed non-coding RNAs that have more than 200 nucleotides, were discovered and implicated in a variety of biological processes [5,6]. In these thousands of lncRNAs, HOTAIR is a star that is highly expressed in primary breast tumors [7], hepatocellular carcinoma [8], colorectal cancer [9] and gastrointestinal stromal tumors [10]. HOTAIR expression is augmented in primary breast tumors and metastases, and HOTAIR expression level in primary tumors is a powerful predictor of metastases and death [7,11].Therefore, HOTAIR may be a potential therapy target in breast cancer. HOTAIR promotes cancer progression in various ways, including dependents EZH2 to promote cell cycle progression [12], regulating PTEN methylation [13] and maintaining the stemness of cancer cells [14]. However, the mechanism by which HOTAIR increases in breast cancer is unknown.

The hormone estrogen (17β-estradiol, E2) has a key role in cell prolife[ration and differentiation through receptor binding and activation [15-17]. The effects of E2 have been widely analyzed in the human mammary gland, where it is responsible for normal epithelial growth and for the development of 70–80% of human breast cancer tumors [18]. Approximately 70% of human breast cancer is estrogen receptor-α positive (ER+) and up to 20% of breast cancer is triple-negative breast cancer (TNBC) [19].

In ER-positive breast cancer, HOTAIR is transcriptionally induced by E2 through multiple functional estrogen response elements (EREs) in the promoter region [20]. However, as a highly aggressive breast cancer subtype, TNBC lacks a known signaling pathway amenable to targeted therapy. G-protein-coupled estrogen receptor-1 (GPER, formerly known as GPR30) has attracted increasing interest, considering its ability to mediate estrogenic signaling in breast cancer [21]. GPER has also been proposed as a candidate biomarker in triple-negative breast cancer [22]. In addition, in our previous study, we found that E2 can regulate miR-148a expression through GPER [23]. Since HOTAIR increases in both ER-positive and TN breast cancer [24,25], we supposed that estrogen may regulate HOTAIR expression through GPER.

To study whether HOTAIR is regulated by E2 via GPER in breast cancer cells, we measured the mRNA levels of HOTAIR in breast cancer cells after treatment with E2. Furthermore, we investigated the regulation mechanism of E2 on HOTAIR expression. We found that E2 up-regulated HOTAIR in breast cancer cells through GPER via the suppression of miR-148a. Taken together, we are reporting a new mechanism of E2 regulating HOTAIR expression in breast cancer.

## Materials and methods

### Patients and sample collection

Tumor and blood samples were obtained from breast adenocarcinoma patients before surgical or other treatment at Zhejiang University Medical School’s Affiliated Second Hospital. Tissue and blood samples were derived from two entirely independent populations. Each patient gave written informed consent. The migration status of tumor was determined by sentinel lymph node biopsy. This study was approved by the Institutional Review Board. The clinicopathologic data are stored in a database in accordance with hospital privacy rules and are summarized in Table 1. All tissue samples were stored in liquid nitrogen within 15 minutes after excision (median delay of 9 minutes). Healthy control tissue was obtained from breast reduction surgery. None of the control samples showed pathological changes. In total, 20 tumor samples and 20 healthy control samples were included.

**Table 1:** **Clinical variables**

Breast cancer variables	
Age (years) median (range)	52.43
Tumor size	
< 2 cm	8
>2 cm	12
Metastasis status	
Metastasizing tumors	10
Non-metastasizing tumors	10
Estrogen receptor status	
Positive	6
Negative	14
Tumor type	
Invasive ductal carcinomas	20
Months surviving (mean)	124

### PBMC isolation

The blood was collected from a cubital vein with an anti-coagulant (heparin sodium) and processed immediately. The controls and patients were matched for age and gender where possible. Peripheral blood mononuclear cells (PBMCs) were separated by centrifugation on Ficoll gradient.

### Cell cultures

MDA-MB-231and BT549 cells were obtained from the Cell Bank of the Chinese Academy of Sciences (Shanghai, China). All cells were maintained in a humidified incubator at 37°C and 5% CO_2_. For the E2 (Sigma-Aldrich, USA) stimulation experiments, the cells were cultured for at least 3 days in phenol red-free RPMI1640 with 5% dextran-coated charcoal-treated serum before the E2 treatment.

### RT-PCR and real-time PCR

RNA was extracted using TRIzol. Total RNA (1 μg) was reverse-transcribed using a RevertAid First Strand cDNA Synthesis Kit (Fermentas). HOTAIR and miR-148a were measured using qRT-PCR (Roche). The expression of HOTAIR was determined in triplicate in three to six separate experiments and normalized using GAPDH, and miR-148a was normalized using U6. Real-time PCR was performed in the ABI PRISM 7300 Sequence Detection System 2.1 (PE Applied Biosystems) using relative quantification. Analysis and fold differences were determined using the comparative cycle threshold (CT) method. Fold change was calculated from the △△CT values with the formula 2^-△△CT^.

The primers are miR-148a -F:5′-ACACTCCAGCTGGGACAAAGTTCTG-3′; miR-148a -R:5′- CTCAACTGGTGTCGTGGAGTCGGCAATTCAGTTGAGTCAGTGCAC -3′; U6 -F:5′-CTCGCTTCGGCAGCACA-3′; U6- R:5′-AACGCTTCACGAATTTGCGT-3′; HOTAIR -F:5′-TTTGGACTGTAAAATATGGC-3′; HOTAIR -R:5′-TTCTGACACTGAACGGACT-3′; GAPDH-F:5′- GTGAAGCAGGCGTCGGA -3′ GAPDH-R:5′- AGCCCCAGCGTCAAAGG -3′.

### RNA oligonucleotides and transfection

The siRNA sequences targeting human HOTAIR (siHOTAIR-1 UAACAAGACCAGAGAGCUGUU; siHOTAIR-2 CCACAUGAACGCCCAGAGAUU; siHOTAIR 3 GAACGGGAGUACAGAGAGAUU) or negative control RNA (NC CUACAACAGCCACAACGUCdTdT) were designed and produced by Genepharma (Shanghai, China). siRNA transfection was performed using Lipo2000 (QIAGEN). siRNAs with 20 nmol/L were used for transfection in a serum-free medium. The total RNA was prepared 24 ~ 48 hours after transfection and used for quantity RT-PCR analysis.

### Migration and invasion assays

For the transwell migration assay, the breast cancer cells were trypsinized and placed in the upper chamber of each insert (Corning, Cambridge, USA) containing the non-coated membrane. Then, a medium supplemented with 20% fetal bovine serum (600 μl) was added to the lower chambers. After 24, 36 and 48 hours of incubation at 37°C, the upper surface of the membrane was wiped with a cotton tip, and the cells attached to the lower surface were stained for 10 min with crystal violet. The cells in five random fields of view at × 100 magnification were counted and expressed as the average number of cells per field of view. All assays were performed in triplicate.

### Immunoblotting

MDA-MB-231 cells were stimulated with 1 μM G1 (Sigma-Aldrich, USA) with or without 100 nM G15 (Sigma-Aldrich, USA) for 6 h. Then cell lysates were harvested in a cell lysis buffer (Boster, Wuhan, China), dissolved in 9% SDS–PAGE buffer, and subjected to western blotting using primary detection antibodies against total or phosphorylated ERK1/2 (diluted 1:1000; BioWorld, St Louis Park, MN, USA). Membranes were incubated overnight at 4°C before incubation with the appropriate HRP-conjugated secondary antibodies. Immunodetection was conducted using the enhanced chemiluminescence system (Amersham Pharmacia Biotech).

### Luciferase reporter assay

The full length of HOTAIR was amplified and cloned into downstream of PGL3-control vector (Promega). Cells plated on 24-well plates were transfected with 100 ng plasmid and 200 nmol/L of miR-148a mimics (RiboBio Co., Ltd., Guangzhou, China), miR-148a inhibitors (RiboBio Co., Ltd., Guangzhou, China) or their negative control (RiboBio Co., Ltd., Guangzhou, China). The miRNA mimics were a sequence of synthetic mature miRNAs used for functional studies of miRNAs. The miRNA inhibitors were synthetic antisense oligonucleotides, which are complementary to the mature miRNA sequence and used for loss-of-function studies of miRNAs. After 48 hours, the cells were lysed and assayed with a dual luciferase assay (Promega) according to the manufacturer’s instructions. For HOTAIR promoter analysis, the HOTAIR promoter (−35 to −2286) was amplified and cloned into a PGL3-basic vector (Promega).Transfection efficiency was estimated by co-transfecting the cells with SV-40 Renilla luciferase. Luciferase activity was measured using the dual luciferase assay system (Promega) and a 96-well luminometer (Fluoroskan Ascent Fl, Labsystems). Three independent experiments were performed in triplicate.

### Statistics

A statistical analysis was performed using Prism 5.0. One-way analysis of variance (ANOVA) and Tukey post hoc tests were used for comparisons within a group. The student t test was used for comparing two different treatments for one cell. All tests were two-sided and p < 0.05 was considered significant. The association analysis was evaluated with Fisher’s exact test.

## Results

### The HOTAIR level was increased in the PBMCs and tumor tissues from the breast cancer patients

**Figure 1:**
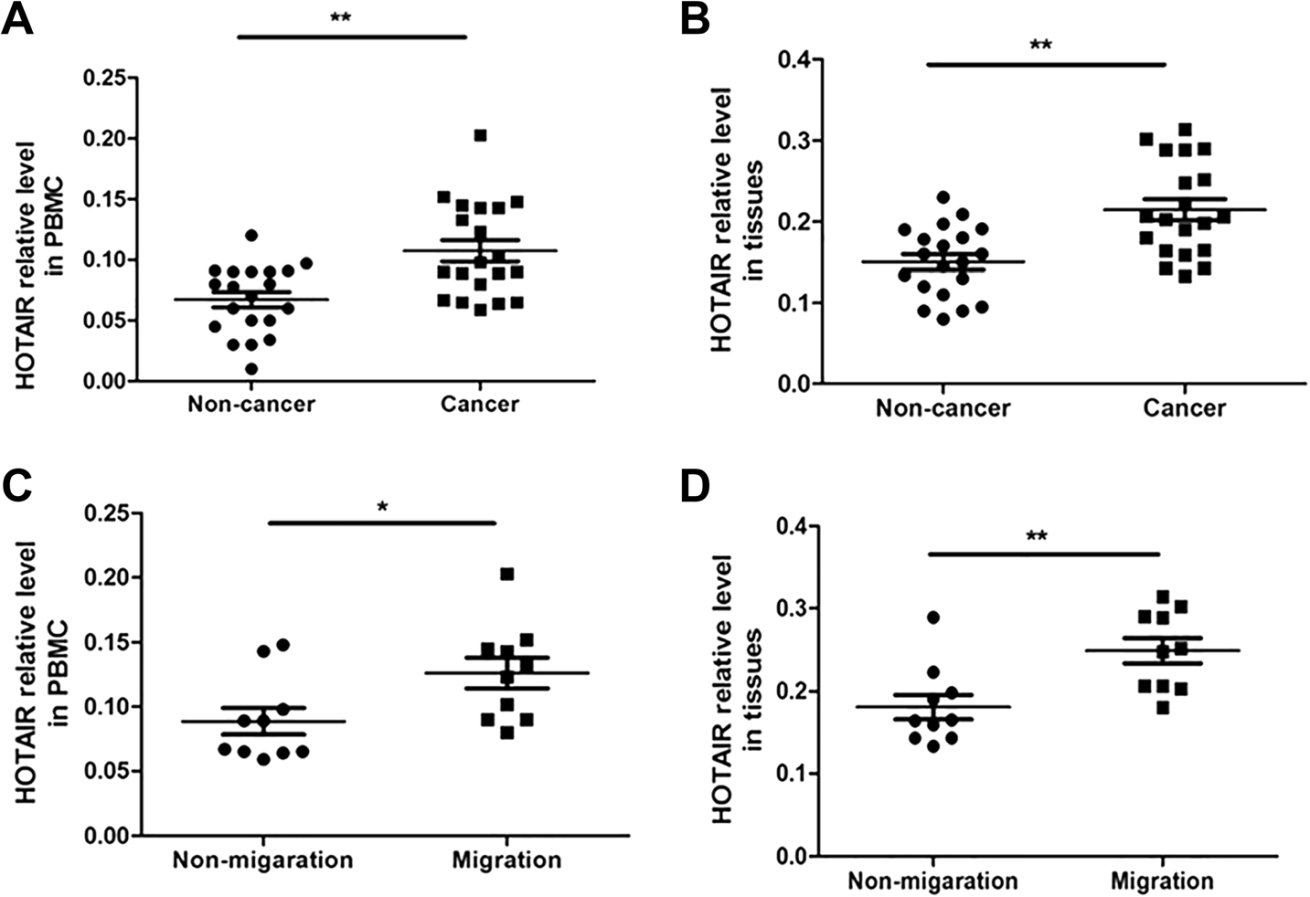
HOTAIR level increased in the PBMCs and tumor tissues from the breast cancer patients. **A)**: PBMCs were collected from 20 patients with breast cancer and 20 healthy women. Then the expression of HOTAIR was detected using real-time PCR. **B)**: Fresh 20 human breast cancer tissues and 20 normal human breast tissues were collected and HOTAIR expression was assessed with real-time PCR. **C)**: PBMCs were collected from 10 patients with breast cancer migration and 10 without migration. Then the expression of HOTAIR was detected using real-time PCR. **D)**: Fresh 10 breast cancer tissues from patients with breast cancer migration and 10 without migration were collected and HOTAIR expression was assessed by using real-time PCR. *p < 0.05, **p < 0.01.

To investigate whether the expression of HOTAIR is changed in breast cancer patients, we collected PBMCs from 20 breast cancer patients and 20 normal women, and measured the HOTAIR levels using real-time PCR. The results showed that the HOTAIR levels in breast cancer were significantly higher than those in normal women (p = 0.0007) (Figure 1A). Moreover, we measured the expression of HOTAIR in breast cancer tissues (n = 20) and normal breast tissues (n = 20). As shown in Figure 1B, HOTAIR was significantly increased in the breast cancer tissues (p = 0.0003). In addition, we also compared the differential expression of HOTAIR in the PBMCs and breast cancer tissues from the patients with migrated breast cancer and non-migrated breast cancer. The results showed that HOTAIR expression was significantly up-regulated in the PBMCs (p = 0.0285) (Figure 1C) and tissues (p = 0.0048) (Figure 1D) from the migrated breast cancer patients.

### E2-induced HOTAIR increases the migration of breast cancer cells

**Figure 2:**
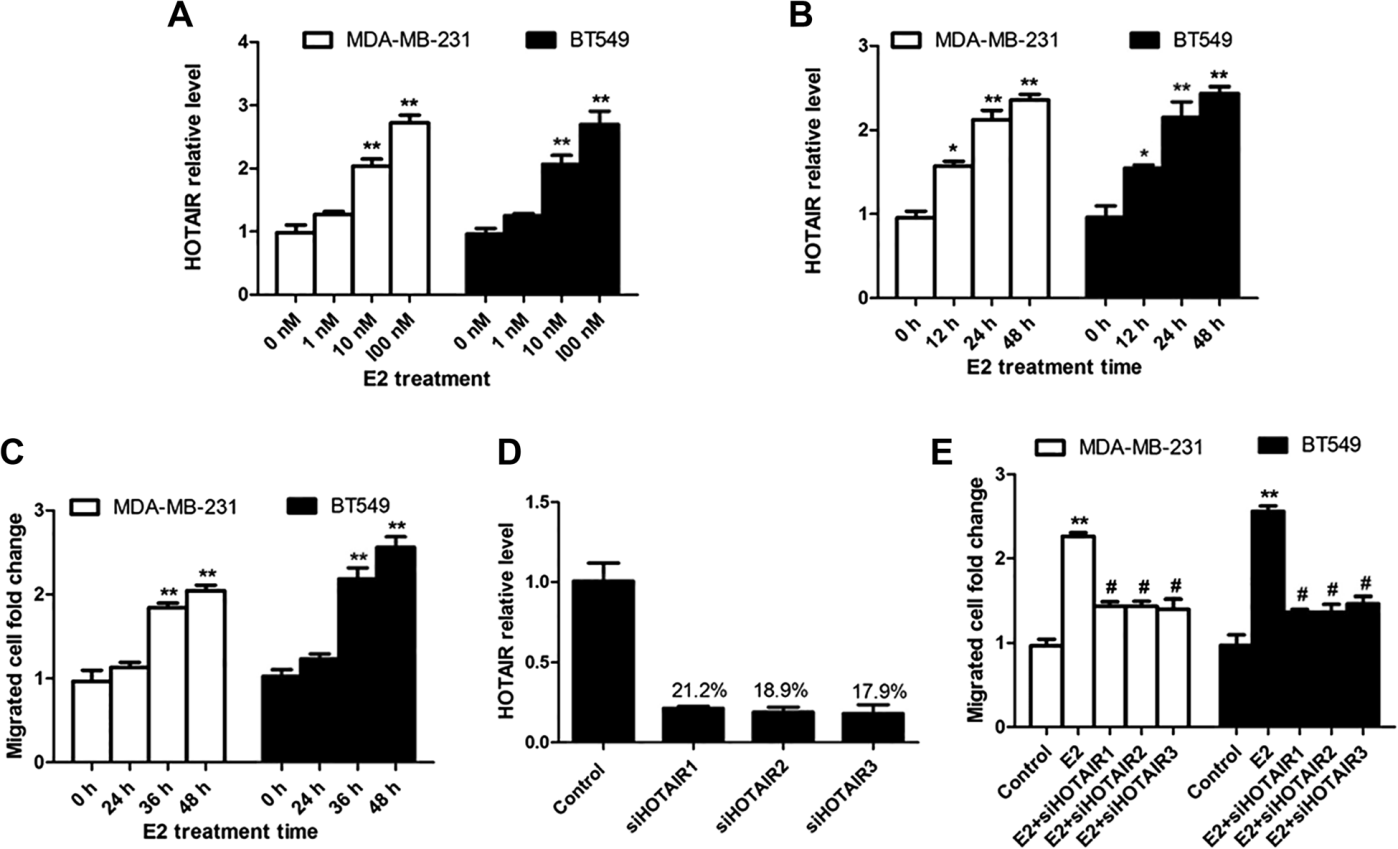
E2-induced HOTAIR increases the migration of breast cancer cells. **A)**: MDA-MB-231 and BT549 cells were treated with the indicated concentration of E2 for 24 hours and the expression of HOTAIR was assessed using real-time PCR. **B)**: MDA-MB-231 and BT549 cells were treated with 10 nM E2 for varying hours and the expression of HOTAIR was assessed using real-time PCR. **C)**: MDA-MB-231 and BT549 cells were treated with 10 nM E2 for varying hours and the migrated cells were counted. **D)**: siHOTAIR RNA sequences (si-HOTAIR1, si-HOTAIR2 and si-HOTAIR3) were transfected into MDA-MB-231 for 24 h, and then the HOTAIR level was detected by using qRT-PCR. **E)**: MDA-MB-231 and BT549 cells were transfected with the control sequence or siHOTAIR RNA sequences before treatment with 10 Nm E2 for 36 hours and the migrated cells were counted. The results are shown as mean ± S.E. from three representative independent experiments. *p < 0.05, **P < 0.01 compared with 0 h or control. ^#^p < 0.05 The results are shown as mean ± S.E. from three representative independent experiments. **P < 0.01 compared with control. ^#^p < 0.05 compared with E2.

To determine whether HOTAIR is a target gene of E2 in triple-negative (TN) breast cancer cells, TN breast cancer cells MDA-MB-231 and BT549 were treated with E2, and HOTAIR expression was measured by using quantitative PCR. HOTAIR expression was significantly up-regulated by E2 in both cell lines (Figure 2A and B). Dose–response experiments revealed maximal HOTAIR reduction with 100 nM after 24 h of treatment in the MDA-MB-231 and BT549 cells, about 2.7 fold of the control group (Figure 2A). Because 10 nM is close to physiological concentration, this concentration is used in the following experiment. Then we detected the E2-induced migration in the breast cancer cells. As shown in Figure 2C, the migration of the MDA-MB-231 (p < 0.01) and BT549 (p < 0.01) cells was significantly increased after treatment with 10 nM E2 for 36 h. Furthermore, we investigated the roles of HOTAIR in E2-induced cancer cell migration. Before E2 treatment, HOTAIR-specific siRNAs (si-HOTAIR1, si-HOTAIR2 and si-HOTAIR3) were transfected into MDA-MB-231 and BT549 cells. The efficiency of the HOTAIR-specific siRNAs was checked with qRT-PCR after transfecting into MDA-MB-231 for 24 h. As shown in Figure 2D, si-HOTAIR1, si-HOTAIR2 and si-HOTAIR3 inhibited the level of HOTAIR to 21.2%, 18.9% and 17.9%, respectively. After deleting HOTAIR with specific siRNAs, the E2-induced cancer cell migration was reversed (Figure 2E).

### GPER mediates the promotion effect of E2 on HOTAIR levels

**Figure 3:**
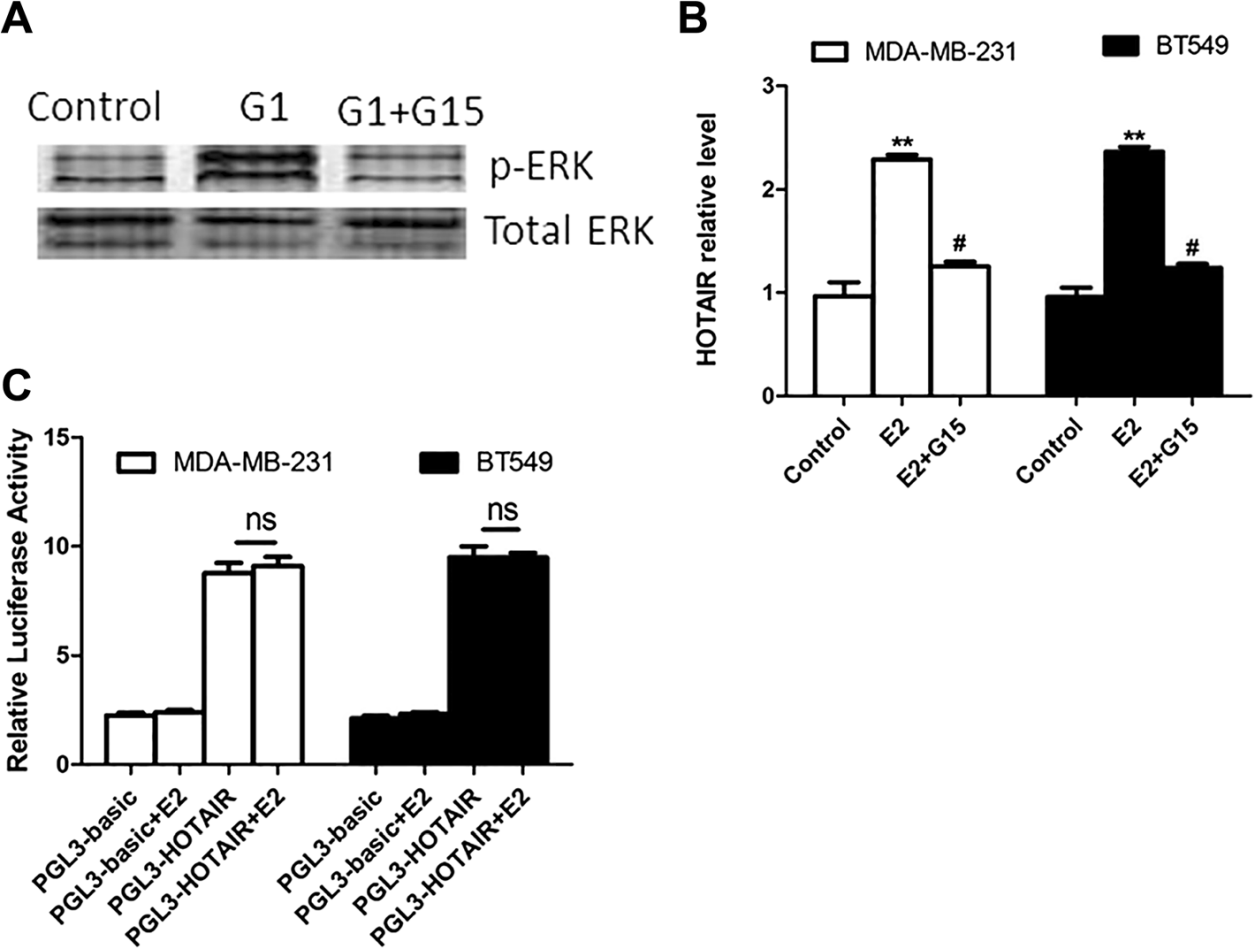
GPER mediates the promotion effect of E2 on HOTAIR expression. **A)**: MDA-MB-231 cells were treated with 1 μM G1 with or without 100 nM G15 for 6 h. Then the expression of the p-ERK level was checked with a western blot. **B)**: MDA-MB-231 and BT549 cells were pretreated with 100 nM G15 for 6 h before the addition of 10 nM E2 for 24 h. Then the expression of HOTAIR was determined by real-time PCR. **C)**: The HOTAIR promoter sequence was cloned into PGL3-basic luciferase reporter plasmid, and then the control plasmid and HOTAIR promoter including plasmid were transfected into the MDA-MB-231 and BT549 cells. Then 10 nM E2 was added into these transfected cells. After E2 treatment for 24 h, the luciferase activity was detected. The results are shown as mean ± S.E. from three representative independent experiments. **P < 0.01 compared with control. ^#^p < 0.05 compared with E2. ns p > 0.5.

Because classical estrogen receptors were negative in the TN breast cancer cells, we speculated that GPER may mediate the effects of E2. First, we checked the inhibition effect of G15 (GPER inhibitor) on GPER signaling. Luo et al. reported that GPER signaling can activate ERK signaling [26]. We treated the MDA-MB-231 cells with GPER agonist G1 (1 μM) with or without 100 nM G15 for 6 h. Then we detected the expression of the p-ERK levels. As shown in Figure 3A, the p-ERK level was increased after G1 treatment, while it was reversed by G15. Then, the MDA-MB-231 and BT549 cells were pretreated with 100 nM G15 for 6 h before E2 treatment. As shown in Figure 3B, E2 can induce HOTAIR levels in both MDA-MB-231 and BT549 cells. However, G15 blocked the E2-induced increase of HOTAIR, indicating that GPER mediated this response. Furthermore, we wanted to investigate whether E2/GPER induce HOTAIR by affecting the HOTAIR promoter. First we constructed the HOTAIR promoter including PGL3-basic luciferase reporter plasmid and transfected them into the MDA-MB-231 and BT549 cells, then 10 nM E2 was added into these transfected cells. After E2 treatment for 24 h, the luciferase activity was detected. As shown in Figure 3C, E2 had no effect on luciferase activity, indicating that E2/luciferase may induce HOTAIR expression in a different way.

### miR-148a targets HOTAIR in breast cancer cells

**Figure 4:**
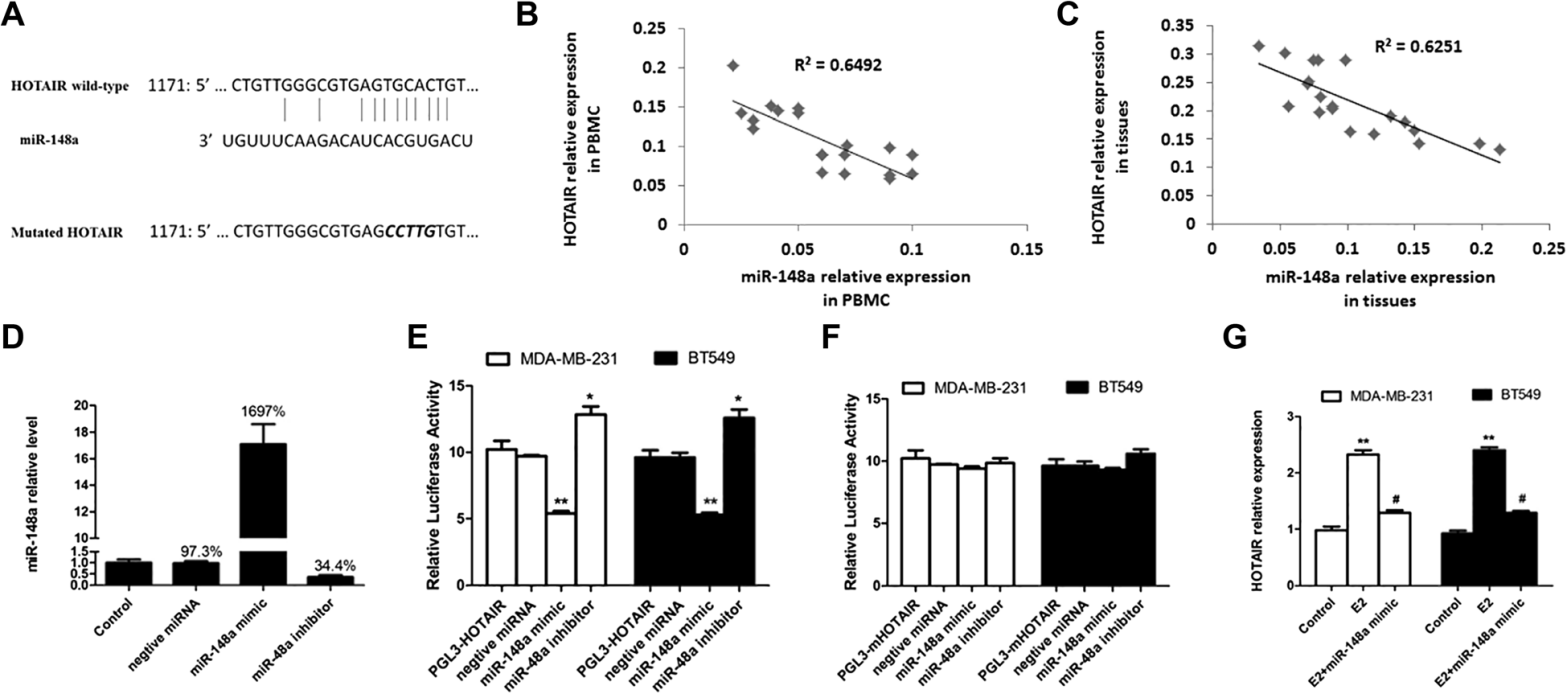
miR-148a targets HOTAIR in breast cancer cells. **A)**: HOTAIR has binding sequences for miR-148a. **B)**: The level of miR-148 was associated with the HOTAIR mRNA level in the PBMCs from the breast cancer patients. Spearman’s correlation coefficient [rs] = r^2^ = 0.6492, p = 0.009. **C**): The level of miR-148 was associated with the HOTAIR mRNA level in the tumor tissues from the breast cancer patients. Spearman’s correlation coefficient [rs] = r^2^ = 0.6251, p = 0.004. **D**: The MDA-MB-231 cells were transfected with negative miRNA, miR-148a mimics and miR-148a for 24 h, and then the level of miR-148a was assessed using qRT-PCR. **E)**: The luciferase activities of the MDA-MB-231 and BT549 cells were measured after co-transfection with PGL3-HOTAIR and miR-148a or its inhibitor for 24 h. The results are shown as mean ± S.E. from three representative independent experiments. **P <0.01 vs. control. **F)**: The luciferase activity of the MDA-MB-231 and BT549 cells was measured after co-transfection with the indicated mutated PGL3-mHOTAIR constructs and miR-148a or its inhibitor for 24 h. The results are shown as mean ± S.E. from three representative independent experiments. **G)**: The MDA-MB-231 and BT549 cells were transfected with miR-148a mimics before E2 treatment. Then the transfected cells were treated with 10 nM E2 for 24 h, and the expression of HOTAIR was checked with real-time PCR. The results are shown as mean ± S.E. from three representative independent experiments. **p <0.01 vs control, ^#^p < 0.05 vs. E2 alone.

It has been reported that lncRNAs can be regulated by miRNAs. We have found that E2 regulated miR-148a expression in breast cancer cells. miR-148a is an anti-migration miRNA in cancer cells. Of note, we predicted that HOTAIR has miR-148a binding sequences using DIANA Tools (http://diana.imis.athena-innovation.gr/DianaTools/index.php?r=lncBase/indexbio) (Figure 4A). Furthermore, we found that the miR-148a level in the PBMCs from the breast cancer patients was also negatively correlated with the HOTAIR level (R^2^ = 0.6492, P < 0.001) (Figure 4B). The miR-148a level in the cancer tissues from the breast cancer patients was also negatively correlated with the HOTAIR level (R^2^ = 0.6251, P < 0.001) (Figure 4C). Next, before the luciferase reporter assay, we checked the transfection efficiency of the miR-148a mimics and miR-148a inhibitors. As shown in Figure 4D, the miR-148a level was 97.3%, 1697% and 34.4% compared to control after transfection with negative miRNA, miR-148a mimics and miR-148a inhibitors, respectively. The luciferase reporter assay demonstrated that miR-148a significantly suppressed the expression of a luciferase reporter gene fused full sequence of HOTAIR, which could be reversed by further introduction of the miR-148a inhibitor in the MDA-MB-231 and BT549 cells (Figure 4E). To further identify that the sequence shown in Figure 4A was miR-148a binding sites, we muted TGCAC (1185–1189) to CCTTG. Then we found that miR-148a could not affect the luciferase activity after the mutated HOTAIR was cloned into the luciferase reporter plasmid (Figure 4F). Furthermore, we found that E2-induced HOTAIR could be reversed by adding miR-148a (Figure 4G).

## Discussion

LncRNAs are of high interest as potential breast cancer therapeutics. However, their expression and function in breast cancer still need to be elucidated. Estrogen signaling is important in the development and progression of breast cancer [15]. HOTAIR, one of the important lncRNAs in the promotion of breast cancer migration, increases in both ER-positive and TN breast cancer [24,25], indicating that estrogen may regulate HOTAIR in a different way other than through ER. In the present study, we found that HOTAIR was increased in breast cancer patients, and was especially higher in migrated breast cancer. In addition, we found that estrogen promoted HOTAIR through its receptor GPER and estrogen-induced breast cancer cell migration was reversed by deleting HOTAIR. Furthermore, we identified that E2-GPER increases the level of HOTAIR through the suppression of miR-148a.

HOTAIR can regulate gene expression through changes in chromatin states and epigenetic modifications [27,28]. Recently, the up-regulation of HOTAIR was observed in several cancers, including breast cancer [7,28-30], hepatocellular carcinoma [11,31], colorectal cancer (CRC) [9,32], pancreatic cancer [33], non-small cell lung cancer (NSCLC) [34] and esophageal squamous cell carcinoma (ESCC) [32,33]. Furthermore, HOTAIR has promoted the migration and invasion of breast carcinoma cells [32], CRC cells [9], pancreatic cancer cells [33], NSCLC cells [34] and ESCC cells [32,33]. Therefore, to investigate the way HOTAIR regulates cells is very important for cancer clinical therapy.

In ER-positive breast cancer cells, it has been demonstrated that HOTAIR is transcriptionally induced by E2 through multiple functional EREs in its promoter [20]. Estrogen receptors (ERs), along with various ER coregulators such as histone methylases mixed-lineage leukemia 1 (MLL1) and MLL3 and CREB-binding protein/p300, bind to the promoter of HOTAIR in an E2-dependent manner. The level of histone H3 lysine-4 trimethylation, histone acetylation and RNA polymerase II recruitment is enriched at the HOTAIR promoter in the presence of E2. The knockdown of ERs and MLLs downregulated the E2-induced HOTAIR expression [20]. However, in TN breast cancer, HOTAIR is also up-regulated, indicating that HOTAIR may be regulated in a different way. Here, we found that E2 could up-regulate HOTAIR levels through GPER in TN breast cancer cells. Our findings further confirm the important role of GPER in cancer development.

E2 regulates the HOTAIR level through the down-regulation of miR-148a. Several miRNAs have been reported to regulate HOTAIR levels. miR-141 suppresses HOTAIR in an Ago2-dependent manner [34]. In addition, HOTAIR can also regulate miRNA levels [35]. miR-148a functions as a tumor suppressor in cancer cells. It has been reported that miR-148a inhibits tumor metastasis by targeting IGF-IR and IRS1 [36]. Moreover, miR-148a suppresses the epithelial-mesenchymal transition and metastasis of hepatoma cells by targeting Met/Snail signaling [37]. We found that the miR-148a level was negatively correlated with the HOTAIR level in breast cancer patients. In addition, we predicted and proved that there are miR-148a binding sequences in HOTAIR. Tumor suppressor miRNA inhibits tumor promoting lncRNA, which may be an important regulation method in cancer.

**Figure 5:**
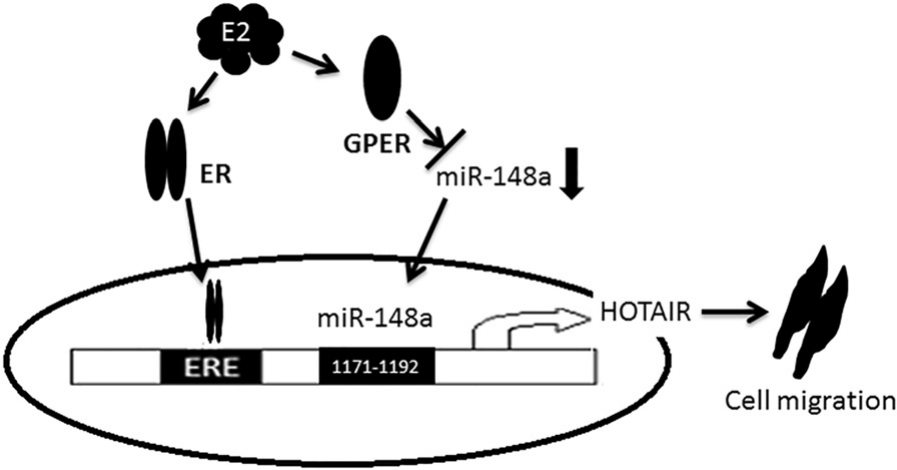
Model of E2-induced breast cancer cell migration via up-regulation of HOTAIR expression.

In summary, HOTAIR level is increased in breast cancer patients and associated with cancer migration. GRER mediates E2-induced HOTAIR levels in breast cancer cells, and E2/GPER promote HOTAIR levels through miR-148a. Therefore, as shown in Figure 5, E2-ER can promote HOTAIR by binding ERE in the promoter of HOTAIR in ER-positive breast cancer. While in TN breast cancer, E2-GPER promotes HOTAIR by inhibiting miR-148a, which can identify the sequence in HOTAIR.

## Conclusions

HOTAIR is becoming a potential therapy target in many cancers. However, its transcription regulation method is unknown. Our findings offer important new insights into the ability of estrogenic GPER signaling to increase the HOTAIR level by inhibiting miR-148a. These findings provide new targets for breast cancer therapy.
